# Tibialis Cranialis Extrafusal and Intrafusal Myofibers and Muscle Spindle Pathology in Canine Degenerative Myelopathy

**DOI:** 10.3390/ani16182817

**Published:** 2026-09-08

**Authors:** Brandie Morgan-Jack, Martin L. Katz, Grace R. Kick, Joan R. Coates

**Affiliations:** 1 Neurodegenerative Diseases Research Laboratory, Department of Ophthalmology, School of Medicine, University of Missouri, Columbia, MO 65212, USA; katzm@health.missouri.edu; 2 Department of Veterinary Pathobiology and Integrative Biomedical Sciences, College of Veterinary Medicine, University of Missouri, Columbia, MO 65211, USA; 3 Department of Pediatrics, School of Medicine Washington University in St. Louis, St. Louis, MO 63110, USA; kick@wustl.edu; 4 Department of Veterinary Medicine and Surgery, College of Veterinary Medicine, University of Missouri, Columbia, MO 65211, USA; coatesj@missouri.edu

**Keywords:** myofiber, skeletal muscle, muscle atrophy, SOD1, dog, amyotrophic lateral sclerosis

## Abstract

Canine degenerative myelopathy (DM) is a progressive, adult-onset neurodegenerative disease, with similarities to superoxide dismutase 1 (*SOD1*)-associated ALS. Clinical signs of DM begin with asymmetric pelvic limb general proprioceptive ataxia and spastic paresis that progresses to flaccid tetraplegia and respiratory failure. Although pelvic limb muscle atrophy and peripheral neuropathy have been reported in DM, intramuscular SOD1 accumulation and muscle spindle pathology remain incompletely characterized. This study evaluated pathological changes in the tibialis cranialis muscle of Pembroke Welsh Corgis with early- and late-stage DM and age-matched unaffected controls. Histologic stains and immunohistochemistry were used to evaluate myofiber morphology, fiber type distribution, muscle spindle integrity, and SOD1 immunoreactivity. Compared to unaffected dogs, DM-affected dogs exhibited increased SOD1 immunolabeling, greater variability in myofiber size, altered myofiber shape and packing, and fibrosis. A progressive reduction in the percentage of type I myofibers was observed without evidence of fiber type grouping. These changes were evident during early disease and became more pronounced with disease progression. Muscle spindles exhibited late-stage disruption of intrafusal myofiber organization and marked increase in SOD1 immunoreactivty. The observed alterations suggest that the muscle pathology was, at least in part, intrinsic to the muscle, rather than being secondary to neurodegeneration. Our findings demonstrate early and progressive pathological changes in the tibialis cranialis muscle, expanding our understanding of skeletal muscle involvement in DM.

## Simple Summary

Canine degenerative myelopathy (DM) is a hereditary disease affecting older dogs. Comparable to some forms of human amyotrophic lateral sclerosis (ALS), DM is characterized by gradual progression of poor balance/loss of coordination, weakness, paralysis, and death. In dogs with DM, the hindlimb muscles are clinically affected early in the course of the disease. This study examined disease-related changes, at the tissue level, in one of these muscles in Pembroke Welsh Corgis with early- and late-stage disease. Compared to unaffected controls, dogs with DM had increased variability in muscle fiber size, changes in the shape and arrangement of muscle fibers, alterations in muscle fiber type composition, and increased scar-like tissue. They also exhibited a buildup, in muscle, of the SOD1 protein that is abnormal in dogs with DM. In late-stage disease, damage was also found in small structures within the muscle known as muscle spindles (structure involved in muscle coordination). These findings further highlight muscle cell abnormalities in DM. Understanding these changes may help explain the loss of coordination and strength experienced by affected dogs and support future research into treatments.

## 1. Introduction

Canine degenerative myelopathy (DM) is a progressive neurodegenerative disorder that has been associated with missense variants in *SOD1* [1,2]. Disease onset typically occurs after 8 years of age and consists of asymmetric general proprioceptive ataxia and spastic paresis in the pelvic limbs [3]. The clinical signs progress to flaccid paraplegia and spread to flaccid tetraplegia, dysphagia and respiratory failure at end-stage disease. Most research on DM has focused on central and peripheral nervous system pathology. However, it is likely that intrinsic muscle pathology contributes to the progressive loss of motor function in this disease [4]. Although the muscle wasting that occurs in DM could be due in part to compromised innervation, it is plausible that the *SOD1* variant has a direct effect on muscle cells. To investigate this, pelvic limb muscles from dogs with early- and late-stage DM were evaluated.

**Figure 1:**
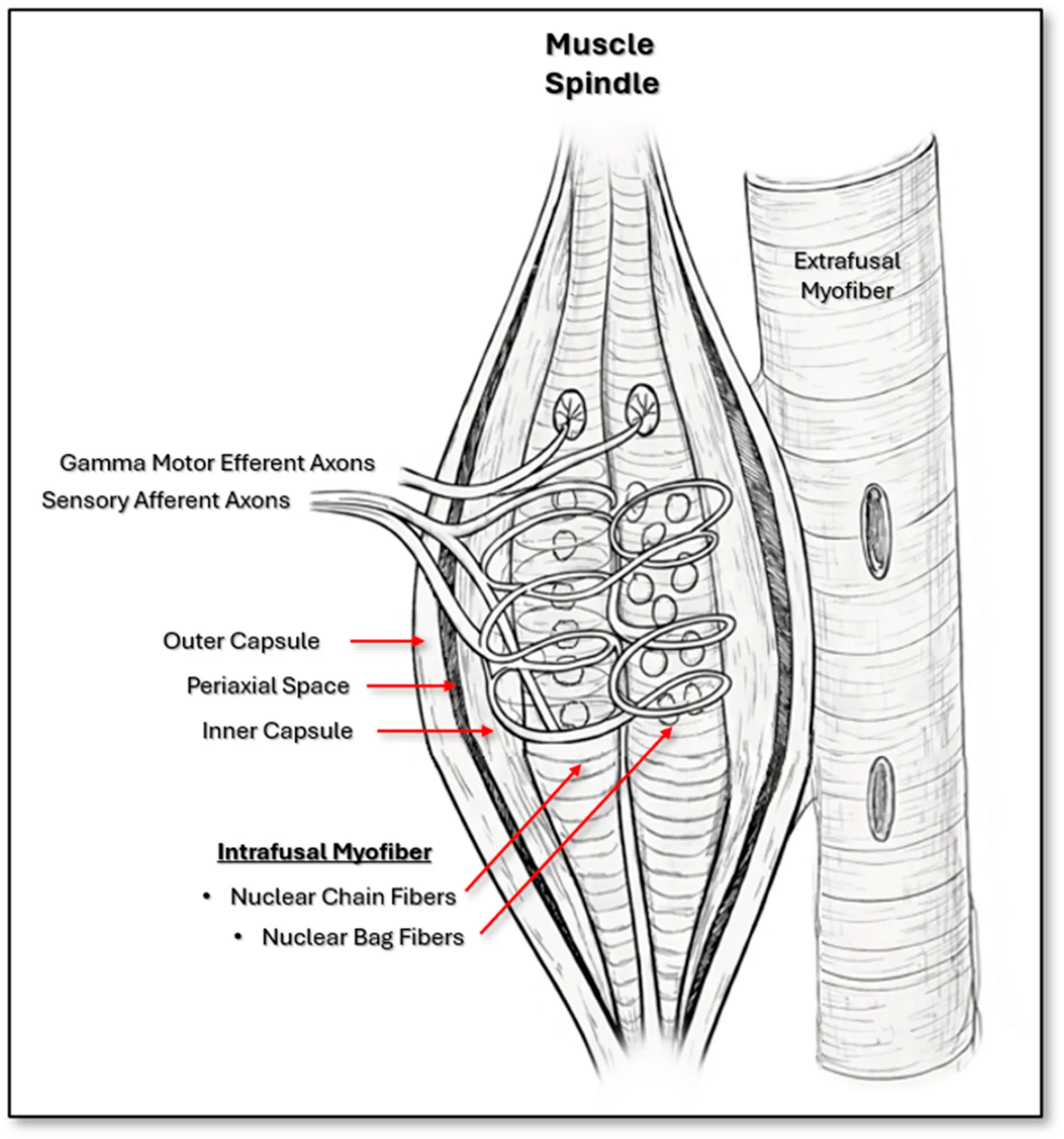
Muscle spindle anatomy: The spindle consists of intrafusal myofibers (nuclear chain and nuclear bag fibers) enclosed within the inner capsule, with a periaxial space separating the inner and outer capsule layers. Muscle spindles run in parallel with extrafusal myofibers and are innervated by sensory afferent axons that relay information on muscle stretch and position to the central nervous system. Gamma motor efferent axons also innervate intrafusal myofibers to regulate spindle sensitivity during muscle contraction (Image created by Brandie Morgan-Jack).

Skeletal muscle contains three primary myofiber populations: type I and type II extrafusal fibers, which generate contractile force, and intrafusal fibers, which function as sensory components of the proprioceptive system (Figure 1). Extrafusal myofibers comprise the bulk of skeletal muscle and are responsible for muscle contraction for gait and stance. In healthy skeletal muscle, these fibers exhibit a relatively uniform polygonal morphology with peripherally located nuclei and are generally classified into slow-twitch oxidative (type I) and fast-twitch glycolytic (type II) fibers based on metabolic and functional properties [5]. Embedded within skeletal muscle are muscle spindles, encapsulated sensory organs composed of specialized intrafusal myofibers arranged parallel to surrounding extrafusal fibers (Figure 1) [6,7]. Each spindle contains several intrafusal fibers organized into nuclear bag or nuclear chain configurations and receives both sensory and motor innervation. Primary Ia and secondary type II afferents detect muscle stretch and length changes, while γ-motor neurons regulate spindle sensitivity [6,7]. Through this arrangement, muscle spindles act as stretch receptors that relay information about muscle length and movement to the central nervous system, conveying proprioceptive information to coordinate appropriate motor control and reflexes [8].

A study was undertaken to test the hypothesis that DM is accompanied by degenerative myofiber pathology in the tibialis cranialis muscle, a pelvic limb muscle clinically affected early in disease progression [9]. We hypothesized that tibialis cranialis muscle from DM-affected dogs would exhibit early and progressive degenerative changes in both extrafusal and intrafusal myofibers and muscle spindles, accompanied by increasing SOD1 accumulation relative to age-matched controls. To test this hypothesis, tibialis cranialis muscles from DM-affected and age-matched unaffected Pembroke Welsh Corgis (PWCs) were evaluated.

## 2. Materials and Methods

### 2.1. Sample Collection and Clinical Classification

Pembroke Welsh Corgis (PWCs) unaffected and affected with DM were enrolled in this study with owner consent, in collaboration with their primary care veterinarians and board-certified veterinary neurologists. Arrangements for collection and donation of tissues were made after contact with JRC. A presumptive diagnosis of DM was made at academic institutions, private specialty hospitals, or general veterinary practices based on clinical signs, disease progression (Table 1), genetic screening for the *SOD1* c.118G > A mutation [1,10], and exclusion of other spinal cord diseases. Based on clinical signs (Table 1), dogs affected by DM were categorized as early-stage (1–2) or late-stage (3–4). Controls were defined as PWCs > 8 years of age and no signs of spinal cord disease; pet owners reached out to JRC for tissue donations.

Tissue samples were collected as soon as possible after euthanasia following standardized protocols. Necropsies at the University of Missouri were performed by a veterinary neurologist (JRC), while external submissions were supported with a tissue collection kit that included all required reagents and instructions for preservation and shipment to the University of Missouri. Offsite tissue collections were performed by primary care veterinarians or veterinary neurologists. A complete list of samples collected at necropsy is provided in Supplemental File S1. Refer to [11] for additional details on sample collection methods. Confirmation of DM diagnosis was based on identification of hallmark histopathologic lesions in the thoracic spinal cord by a veterinary pathologist [4]. All procedures were approved by the University of Missouri Animal Care and Use Committee.

**Table 1:** Neurological signs present at time of euthanasia used to classify disease stage among dogs with DM.

Disease Stage *	Neurological Signs
1 (Early-stage)	Asymmetric general proprioceptive ataxia and spastic paresis in pelvic limbs Intact spinal reflexes
2 (Early-stage)	Non-ambulatory paraparesis, paraplegia Reduced to absent pelvic limb spinal reflexes Pelvic limb muscle atrophy ±Urinary/fecal incontinence
3 (Late-stage)	Flaccid paraplegia, thoracic limb paresis Absent spinal reflexes Severe pelvic limb muscle atrophy Urinary/fecal incontinence
4 (Late-stage)	Flaccid tetraplegia Absent spinal reflexes Severe generalized muscle atrophy Urinary/fecal incontinence Dysphagia, dysphonia, respiratory difficulty

* Dogs categorized as stages 1–2 were grouped as the early-stage cohort, and those classified as 3–4 were grouped as the late-stage cohort [3,12].

### 2.2. SOD1 Immunohistochemistry

A proximal portion of the tibialis cranialis muscle was dissected from each dog. A slice from each sample was fixed in a solution consisting of 3.5% paraformaldehyde (Electron Microscopy Sciences, catalog # 15714, Morgantown, PA, USA), 0.05% glutaraldehyde (Electron Microscopy Sciences, catalog # 16320, Morgantown, PA, USA), 120 mM sodium cacodylate (Electron Microscopy Sciences, catalog # 12310, Morgantown, PA, USA), and 1 mM CaCl2 (Fisher Scientific, catalog # BP510-500, Waltham, MA, USA), pH 7.4, and embedded in paraffin. Deparaffinized sections of each of the samples were then immunolabeled for SOD1 localization as described previously [4,12]. The primary antibody used for immunolabeling was anti-SOD1 (Abcam, catalog #ab13598, Waltham, MA, USA) [1:100]. The dependence of the labeling on the presence of the antibody was confirmed by treating adjacent tissue sections with the same labeling procedure, but with the primary antibody replaced with non-immune serum.

### 2.3. Muscle Fiber Morphology and Muscle Fibrosis

A second piece of the muscle sample was fixed in 2.5% glutaraldehyde (Electron Microscopy Sciences, catalog # 16320, Morgantown, PA, USA) and 0.1 M sodium cacodylate (Electron Microscopy Sciences, catalog # 12310, Morgantown, PA, USA), pH 7.4, at room temperature. After incubating the sample for a minimum of 48 h in this solution, a piece of the muscle approximately 5 mm × 8 mm × 3 mm was dissected, post-fixed in osmium tetroxide (Electron Microscopy Sciences, catalog # 19150, Morgantown, PA, USA) and embedded in epoxy resin (Electron Microscopy Sciences, catalog # 14121, Morgantown, PA, USA). Sections of each muscle were cut at thicknesses of 0.25–0.4 µm with the muscle fibers oriented in cross-section. The sections were mounted on glass slides, stained with Toluidine blue (Electron Microscopy Sciences, catalog # 14950, Morgantown, PA, USA), and imaged with a 20× objective with a Leica DMI 6000B microscope (Leica DMC6200 camera (Leica, Teaneck, NJ, USA). For each sample, tiled images spanning the entire muscle cross-section were acquired and merged to generate a composite image (one tissue section per dog). Approximately 10 non-adjacent fields distributed throughout the cross-section were selected for analysis, to provide representative sampling across the tissue. Each field contained approximately 10–15 myofibers, allowing a minimum of 100 myofibers to be measured per sample. Each muscle fiber was manually outlined using Adobe Photoshop (Adobe, Inc., San Jose, CA, USA). The areas enclosed by each outline were then measured using Image J (National Institute of Health, Bethesda, MD, USA, version 1.47t). Myofibers that extended beyond the selected field of view, preventing clear delineation of their boundaries, were not included in the analysis. The marking and measurement of muscle fibers in each image were performed in a masked manner [4]. Muscle fibrosis was evaluated qualitatively with Masson Trichrome staining using standard methods (Supplemental File S2).

### 2.4. Muscle Fiber Type Analysis Methods

A third proximal portion of the tibialis cranialis muscle from each dog was placed immediately in 0.17 M sodium cacodylate (Electron Microscopy Sciences, catalog # 12310, Morgantown, PA, USA), pH 7.4, at room temperature. Each sample was dissected to obtain a piece that had at least one face at which the muscle fibers were primarily in cross-sectional orientation. The samples were embedded in paraffin, and sections were cut at a thickness of 5 µm. The deparaffinized sections were then immunostained with an anti-myosin heavy chain type 1 (MHC-1) antibody to selectively label type 1 fibers. Labeling was performed using the intelliPATH FLX^®^ (BioCare Medical, Pacheco, CA, USA). Sections were blocked in 3% hydrogen peroxide for 15 min, pretreated with Proteinase K for 5 min (Cell Signaling technology; Catalog #S3020 Danvers, MA, USA), blocked with Background Sniper (Biocare medical; BS966H) for 10 min, incubated with primary antibody MHC-1 (Abcam; catalog #ab11083, Waltham, MA, USA) for 30 min, incubated with MACH 2 Universal HRP Polymer Detection (Biocare medical) for 30 min, developed with Romulin Red (Biocare medical; RAEC810M) for 10 min, and counterstained with hematoxylin (Biocare medical; IPCS5006) for 5 min. The immunolabeled sections were imaged with a Leica DMI 6000B or equivalent, and the numbers of stained and unstained fibers were manually counted in each section. A minimum of 400 muscle fibers from each sample were evaluated. Fiber type analysis was performed in a masked manner.

### 2.5. Statistical Analyses

Quantitative data for this study were collected from unaffected dogs >8 years of age as controls and from early- and late-stage DM dogs. In cases where the early disease sample sizes were too limited for reliable statistical analysis, comparisons were restricted to control and late disease groups using Student’s *t*-test. For all other outcomes, a one-way ANOVA was conducted to assess overall group differences. When the ANOVA indicated significance, post hoc pairwise comparisons were carried out using the Holm–Sidak test. All statistical tests were conducted using Sigma Plot (Version 16.0, Systat Software, Inc., San Jose, CA, USA).

## 3. Results

### 3.1. Classification of Signalment, Genetic Testing and Disease Stage

A total of nineteen PWCs were evaluated in this study. The age at euthanasia, sex, neuter status, and *SOD1* genotype for each dog are provided in Table 2. The mean age at which the dogs were euthanized are as follows: control dogs—13.8 years (median age: 14.0; age range: 10.8 to 15.8 years); early-stage DM—12.7 years (median age: 12.6; age range 12.1 to 13.3 years); late-stage DM—13.0 years (median age: 13.1; age range 11.0 to 15.6 years). ANOVA analysis indicated that there were no significant differences between the groups in the ages at euthanasia (*p* = 0.439). The study included 11 female and eight male dogs. The control group consisted of five female dogs, with all but one spayed. The early-stage group consisted of three spayed females, and three castrated males. The late-stage group consisted of three females (one spayed) and five castrated males.

All except one dog included in this study was homozygous for the *SOD1* c.118A variant associated with DM (Table 2). The one exception was an unaffected dog euthanized at 15.8 years of age that tested heterozygous for the risk variant. Fourteen dogs had signs of DM. six of the dogs were in early-stage DM at the time of euthanasia. The remaining eight affected dogs had signs of late-stage DM. Histopathology on the thoracic spinal cord of all affected dogs confirmed lesions characteristic of DM [3,4,13]. Five PWCs were included as controls and had normal spinal cord on histopathology [4].

### 3.2. Muscle Fiber Size and Morphology

**Figure 2:**
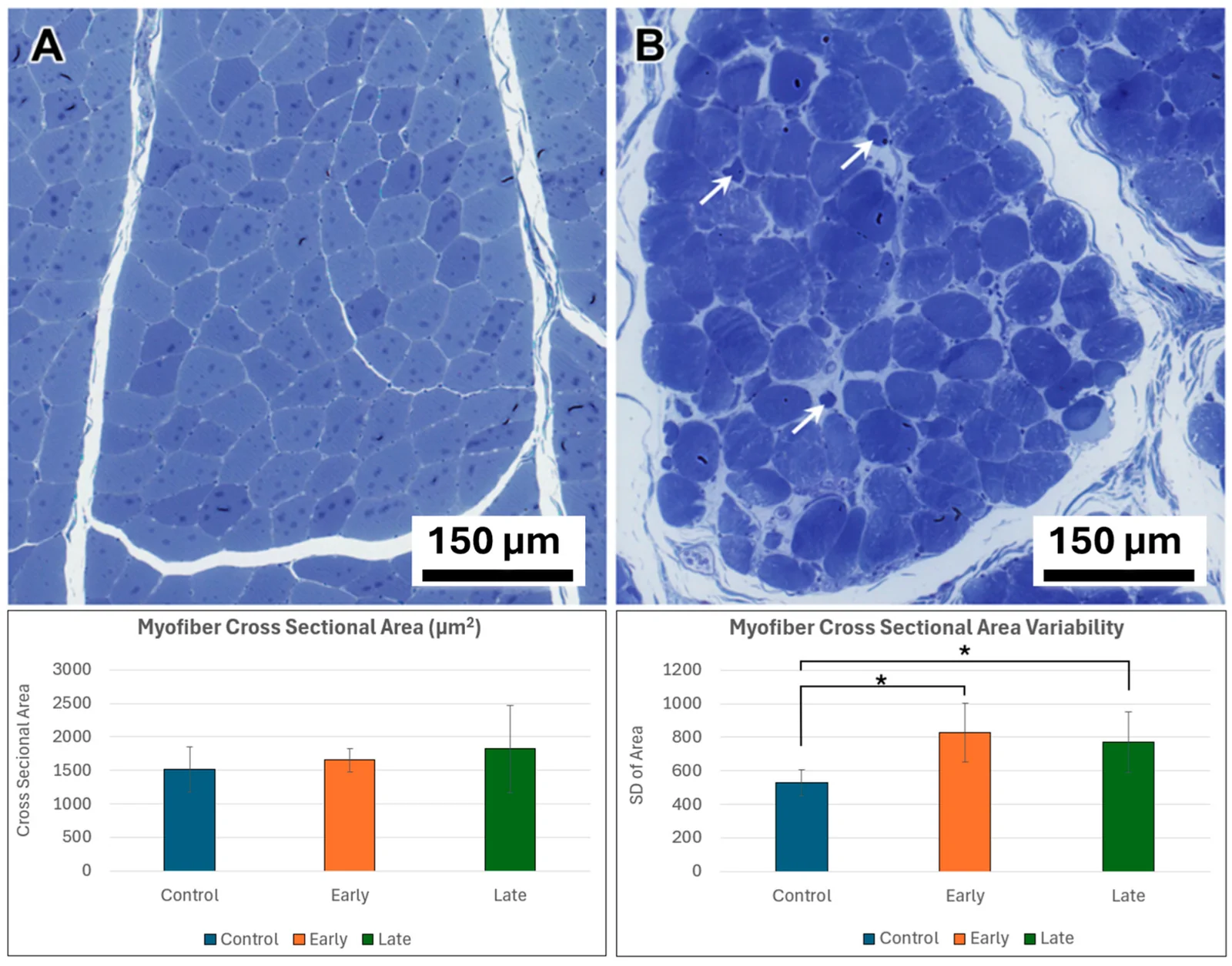
Increased variability in DM-affected myofiber sizes. Light micrographs of Toluidine blue-stained cross-sections of muscle tibialis cranialis from an 11-year-old unaffected PWC (**A**) and from a 13.9-year-old PWC with late-stage DM (**B**). Small atrophic fibers were present in the muscles from affected dogs (arrows in (**B**)). Bar graphs display (**left**) mean myofiber cross-sectional area and (**right**) variability measured by standard deviation (SD) between groups. No significant differences were observed in mean fiber cross-sectional area between groups. However, variability (SD) was significantly increased in early-stage DM (*p* = 0.01) and late-stage DM (*p* = 0.02) compared to controls (denoted by the *), with no significant differences between early- and late-stage groups. Control (*n* = 5), Early (*n* = 5), Late (*n* = 7).

**Figure 3:**
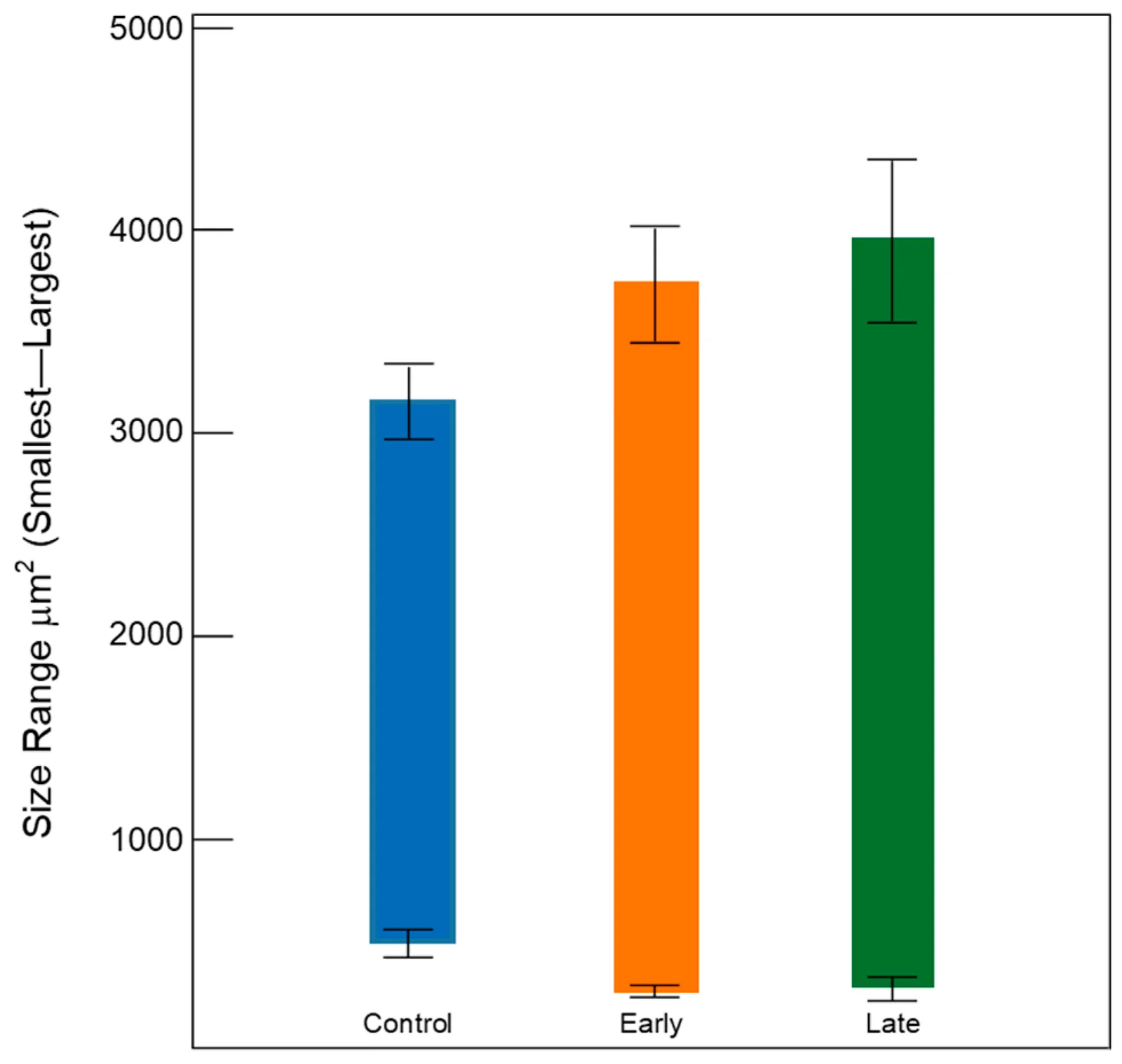
Increased range in DM-affected myofiber sizes. The mean minimal and maximal muscle fiber cross-sectional areas were significantly greater in DM-affected dogs at both early- and late-stage disease than in age-matched dogs that did not exhibit signs of DM (*p* < 0.05).

**Figure 4:**
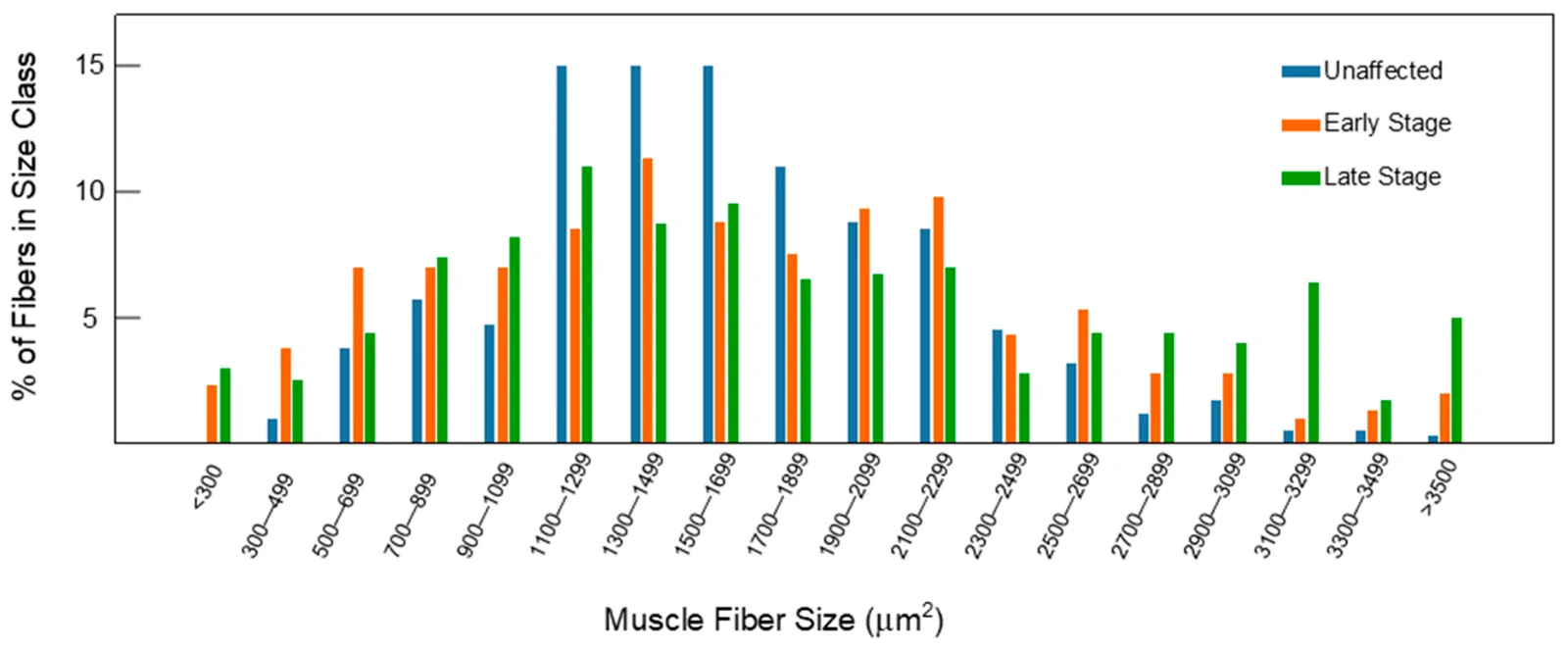
Muscle fiber cross-sectional area size distribution in tibialis cranialis muscles from dogs with early- and late-stage DM and age-matched unaffected dogs. In both early- and late-stage dogs, proportions of fibers less than 1100 and greater than 2500 μm^2^ in cross-sectional area were increased relative to the control dogs. Data shown represent the mean cross-sectional areas determined from all the dogs in each disease status group.

There were no significant differences in mean tibialis cranialis fiber cross-sectional area between dogs with early- or late-stage DM and control dogs that had not exhibited any signs of DM by the time of euthanasia (Figure 2). However, the DM-affected dogs exhibited significantly greater variability in muscle fiber cross-sectional area than did the age-matched control animals. This increased variability was already evident in dogs with early-stage disease. This variability was due to the presence of small atrophic fibers, as well as fibers with increased cross-sectional areas in the muscles of affected dogs at both early- and late-stage disease and included (Figure 2, Figure 3 and Figure 4). Both the abnormally small- and large-diameter fibers were primarily ovoid in profile.

**Figure 5:**
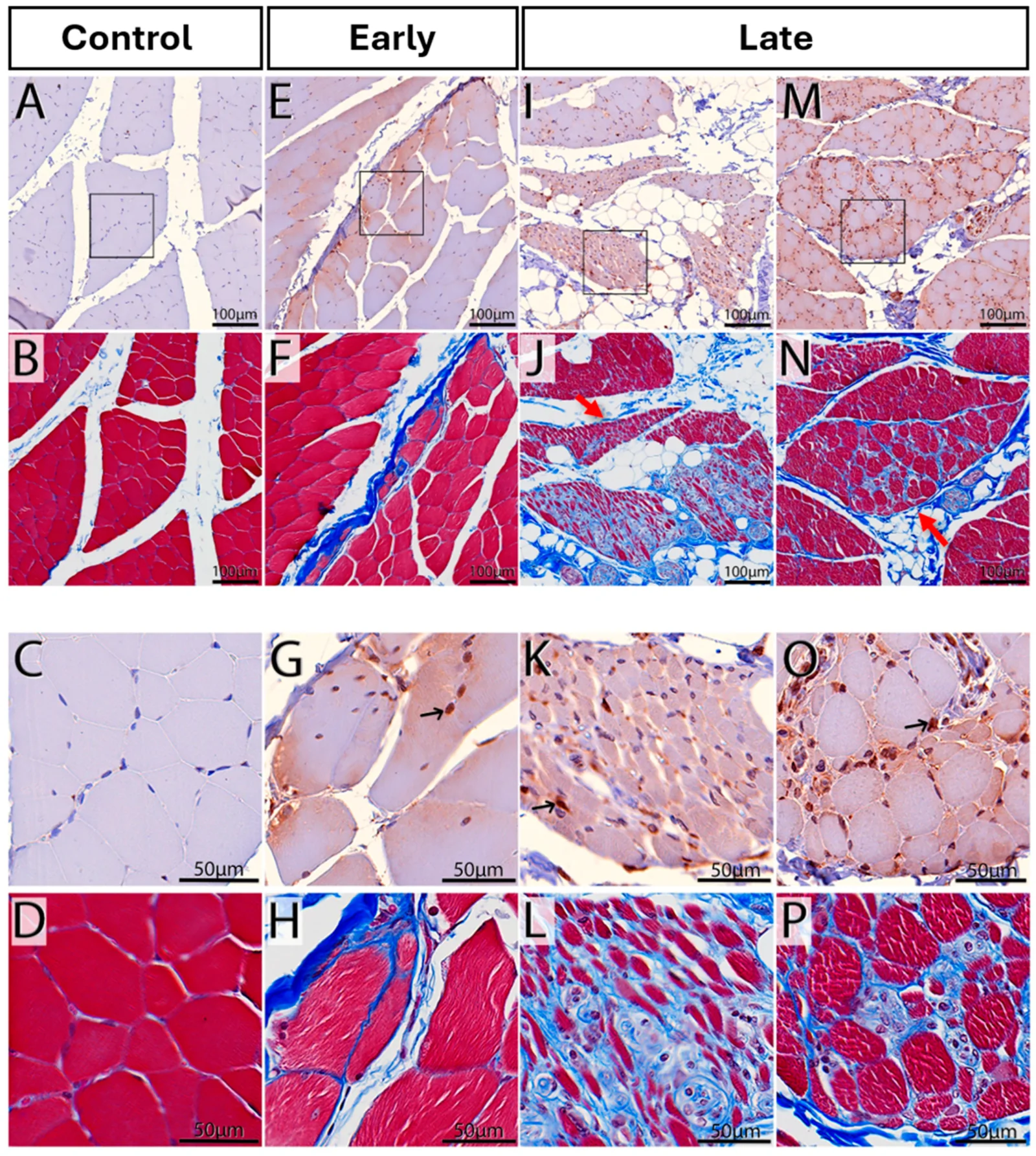
Progressive increase in SOD1 immunolabeling and fibrosis in tibialis cranialis muscle of dogs with DM. Low-magnification images (rows 1–2) and higher-magnification views of boxed regions (rows 3–4) from control (**A**–**D**), early-stage DM (**E**–**H**), and late-stage DM (**I**–**P**) muscles. Rows 1 and 3: SOD1 labeling (SOD1 positive labeling is reddish brown; sections are counterstained with hematoxylin which label nuclei deep blue/purple); Rows 2 and 4: Masson trichrome staining (red= myofibers, blue =collagen). Control muscle shows minimal SOD1 labeling and tightly packed myofibers. Early-stage DM exhibits localized SOD1 immunolabel with emerging fibrosis (blue). Late-stage DM demonstrates widespread SOD labeling and marked collagen expansion. Two regions from the same late-stage muscle illustrate heterogenous pathology. Areas of diffuse myofiber atrophy were observed alongside regions of pronounced variation in myofiber size distribution (red arrows in (**J**) and (N), respectively). Black Arrows: small cells with robust SOD1 labeling. Micron bars = 50 µm.

In addition to the increased variability in muscle fiber cross-sectional area, DM was associated with significant changes in muscle fiber shape and packing density. In the control dogs, the muscle fibers were tightly packed and polygonal in shape. In the affected dogs, there was significantly more space around individual muscle fibers, which were more rounded in shape. These changes were already apparent, but less pronounced, in dogs euthanized in early-stage DM. Masson Trichrome results parallel the increased morphological changes observed in affected muscles. Early-stage samples contained focal regions of increased collagen deposition. By late-stage disease, collagen expansion was more diffuse, with most fascicles exhibiting large areas of perimysial and endomysial collagen surrounding myofibers (Figure 5). These progressive alterations in myofiber morphology and fibrosis highlight substantial structural remodeling of the muscle as DM advances.

### 3.3. SOD1 IHC and Masson Trichrome Staining

**Figure 6:**
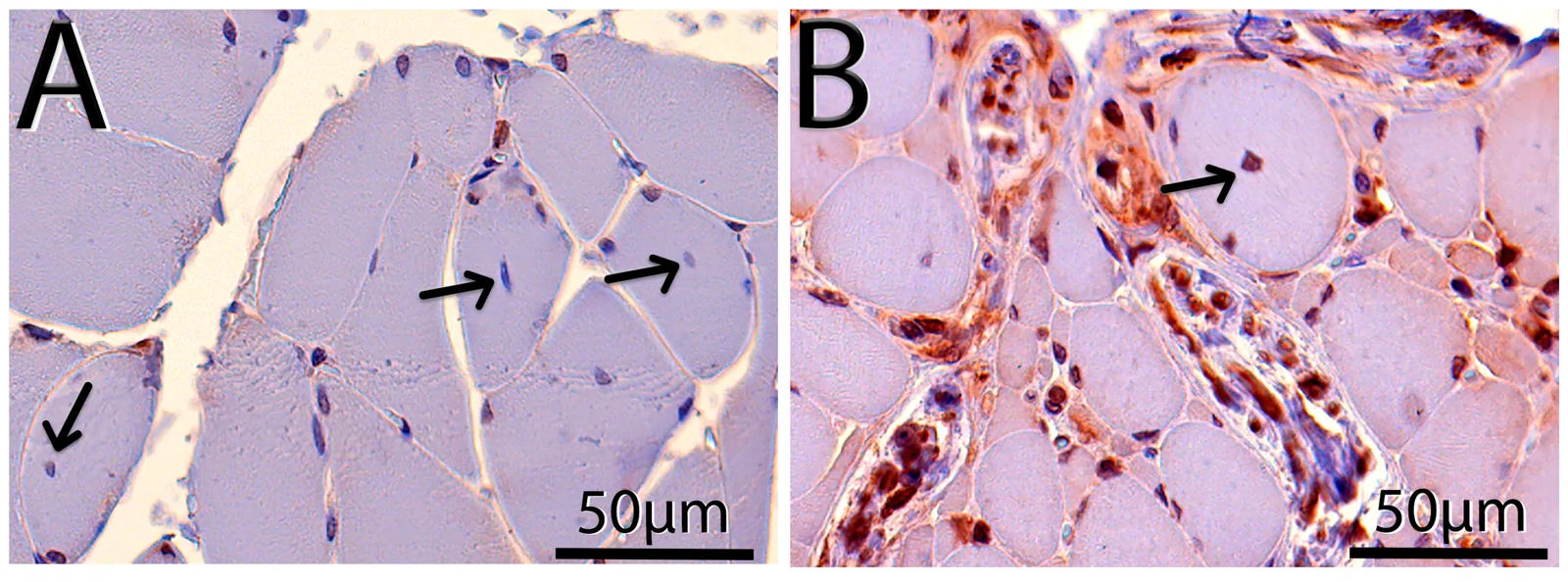
Internalized nuclei observed in DM-affected tibialis cranialis muscle. SOD1-immunolabeled sections from early-stage (**A**) and late-stage (**B**) DM dogs demonstrated centrally located nuclei (arrows), indicative of myofiber remodeling. SOD1 positive labeling is reddish brown; sections are counterstained with hematoxylin which label nuclei deep blue/purple. Micron bar = 50 µm.

SOD1 immunolabeling in the tibialis cranialis muscle differed markedly across disease stages (Figure 5). The labeling pattern in affected myofibers was consistent with abnormal intracellular SOD1 accumulation, while unaffected control dogs exhibited little to no detectable SOD1 labeling within myofibers. Dogs with early-stage DM showed focal areas with strong SOD1 immunoreactivity. Within each muscle cross-section, these early changes were heterogeneous, with labeling intensity sometimes varying among myofibers within the same fascicle. Notably, SOD1-enriched regions frequently corresponded with areas containing increased collagen deposition (Figure 5). In late-stage samples, SOD1 labeling was robust and widespread, involving the majority of myofibers. In late-stage dogs, multiple patterns of muscle degeneration were observed. Within the same muscle cross-section, some regions exhibited features consistent with denervation atrophy, including clusters of degenerating fibers, while other fascicles showed a more widespread generalized atrophy. Additionally, internalized myonuclei were present in both early- and late-stage samples (Figure 6).

**Figure 7:**
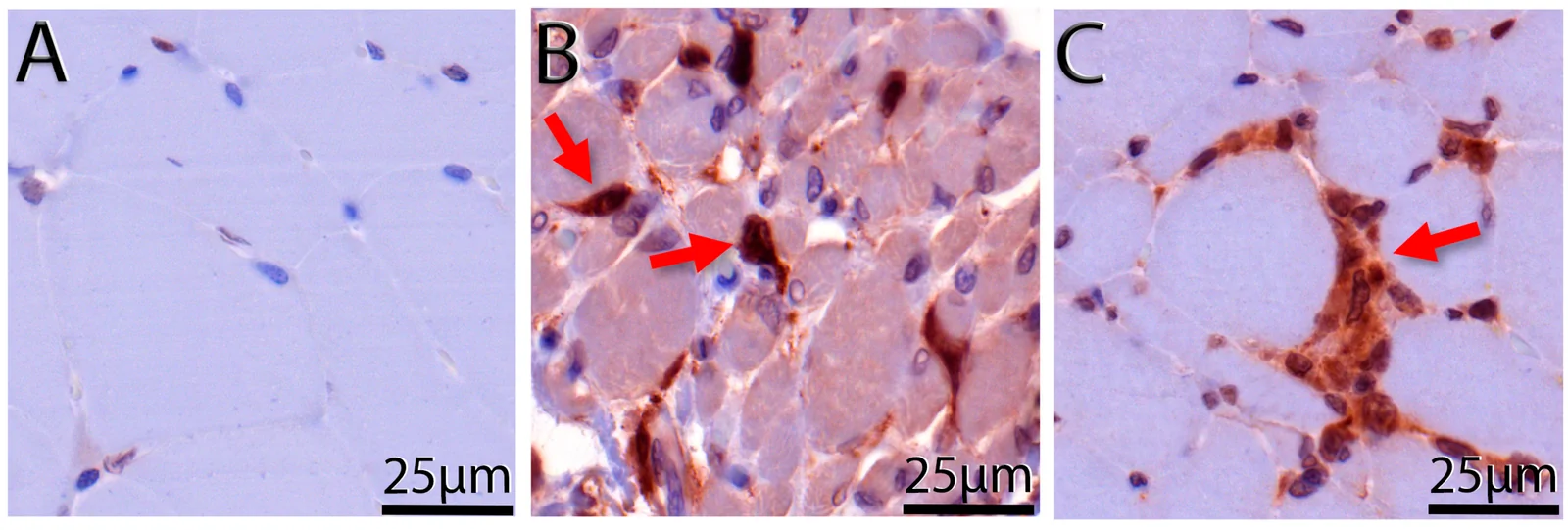
SOD1 labeling in cells located between myofibers. (**A**) Control muscle showing minimal immunoreactivity. (**B**,**C**) Late-stage DM sample with strong SOD1 labeling in numerous cells located between myofibers (arrows), indicating that SOD1 localization is not restricted to myofibers. (SOD1 positive labeling is reddish brown; sections are counterstained with hematoxylin which label nuclei deep blue/purple). Micron bar = 25 µm.

In addition to SOD1 immunoreactivity within myofibers, numerous small SOD1-positive cells were observed within the space between myofibers (Figure 5, Figure 6 and Figure 7). Similar SOD1-positive cells were present in control samples, but were relatively sparse and scattered throughout the tissue. The locations and sizes of the labeled cells are consistent with those of satellite cells, fibroadipogenic progenitors, mesenchymal stromal cells, and macrophages. These interstitial cell types cannot be distinguished based on their location or size. In early-stage samples, these SOD1-positive cells were observed more frequently and were often localized in regions containing extrafusal myofibers with strong SOD1 immunoreactivity. In late-stage DM samples, these cells appeared to be more numerous and distributed throughout the sample. Morphically, these cells were small with sparse cytoplasm, or slightly larger and irregularly shaped. Overall, these findings indicate that aberrant SOD1 deposition begins during early disease and becomes progressively more widespread, ultimately involving most myofibers and numerous supporting cells in late-stage DM.

Masson trichrome staining showed areas of pronounced fibrosis in late-stage disease, with diffuse collagen deposition both within and between fascicles (Figure 5). Aberrant collagen deposition was also apparent in early-stage disease, but was present primarily between fascicles. In late-stage disease, some areas of the muscle also showed large increases in the interstitial space between fascicles that had foamy appearance (Figure 5).

### 3.4. Muscle Fiber Type

**Figure 8:**
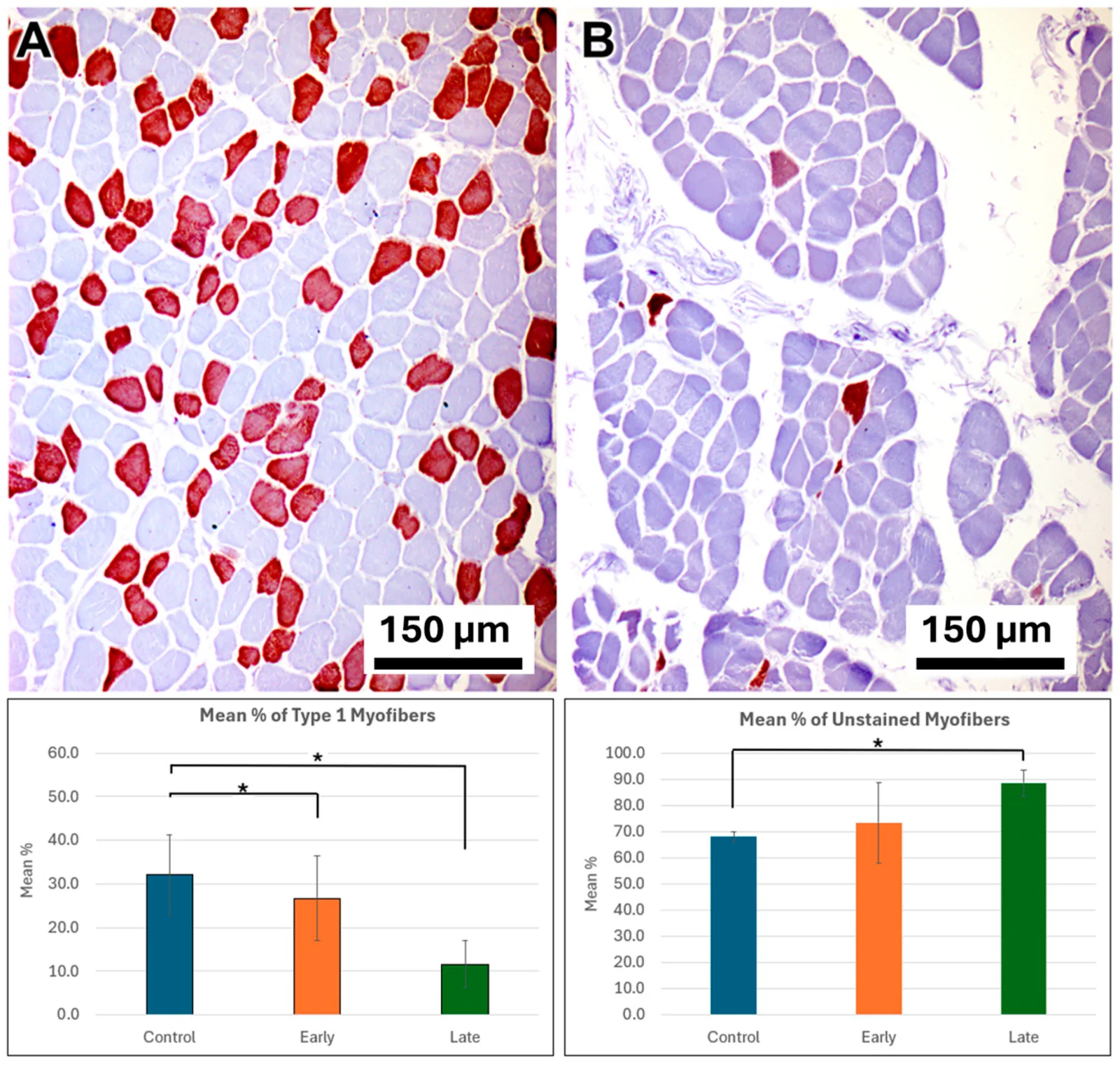
Changes in fiber type predominance. Light micrographs of cross-sections of tibialis cranialis muscle from a 13.5-year-old unaffected PWC (**A**) and from a 15.6-year-old PWC with late-stage DM (**B**). Samples were preserved in cacodylate buffer, and the sections were immunostained with an antibody that labels type I fibers (brownish-red color). The proportion of the muscle fibers that were type I was substantially lower in DM-affected dogs. Bar graphs: (**Left**) shows a significant reduction in type I myofibers in early-stage (*p* = 0.046) and late-stage (*p* = 0.002) (denoted by the *) DM compared to controls, with no significant differences between early- and late-stage groups. (**Right**) indicates that there were significantly more unstained myofibers in late-stage samples compared to controls (*p* = 0.028). Control (*n* = 4), Early (*n* = 5), Late (*n* = 6).

Among unaffected dogs, an average of approximately 36% of the fibers in the muscle tibialis cranialis were type I (Figure 8). In early-stage samples, a significantly lower average proportion of only about 22% of the fibers were type I (Figure 8). Among the dogs with late-stage DM, an average of only 11.8% of the muscle fibers were type I. Unstained myofibers (presumptive type II myofibers) accounted for averages of 67.8%, 72.1%, and 88.2% of control, early- and late-stage samples, respectively. There were significantly more unstained myofibers in late-stage samples compared to control (Figure 8). DM did not result in fiber type grouping that is characteristic of motor neuron compensatory reinnervation.

### 3.5. Muscle Spindle

**Figure 9:**
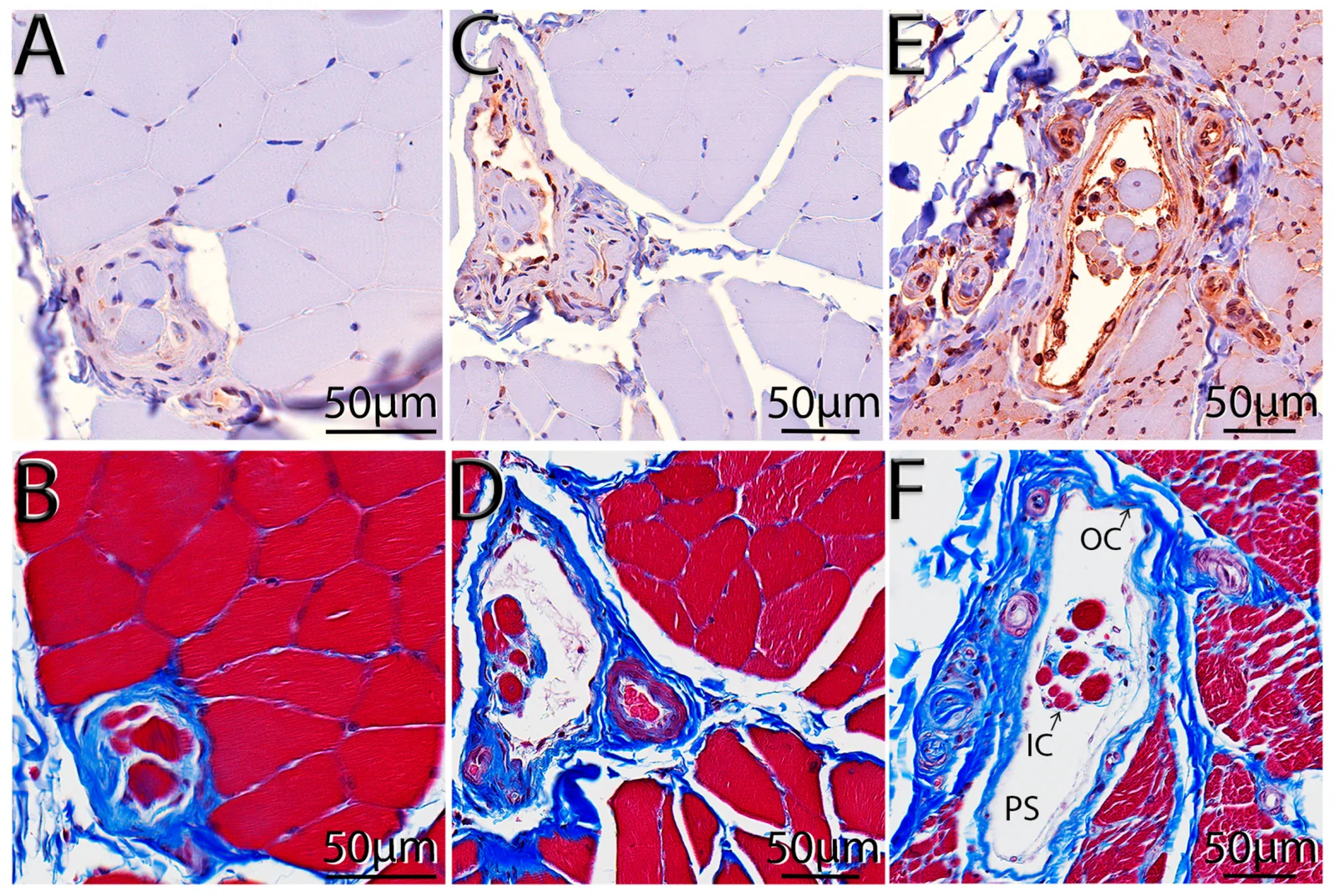
Progressive muscle spindle pathology in DM cranial tibialis muscle. (**A**,**B**) Control spindle showing tightly packed intrafusal myofibers and intact capsule layers with minimal SOD1 labeling. (**C**,**D**) Early-stage DM spindle with increased SOD1. (**E**,**F**) Late-stage DM spindle showing marked SOD1 immunoreactivity and disruption of intrafusal fiber organization. Top row: SOD1 immunohistochemistry (SOD1 positive labeling is reddish brown; sections are counterstained with hematoxylin which label nuclei deep blue/purple).; bottom row: corresponding Masson trichrome staining (red= myofibers, blue =collagen). OC= outer capsule; IC = inner capsule; PS = periaxial space. Micron bars = 50 µm.

**Figure 10:**
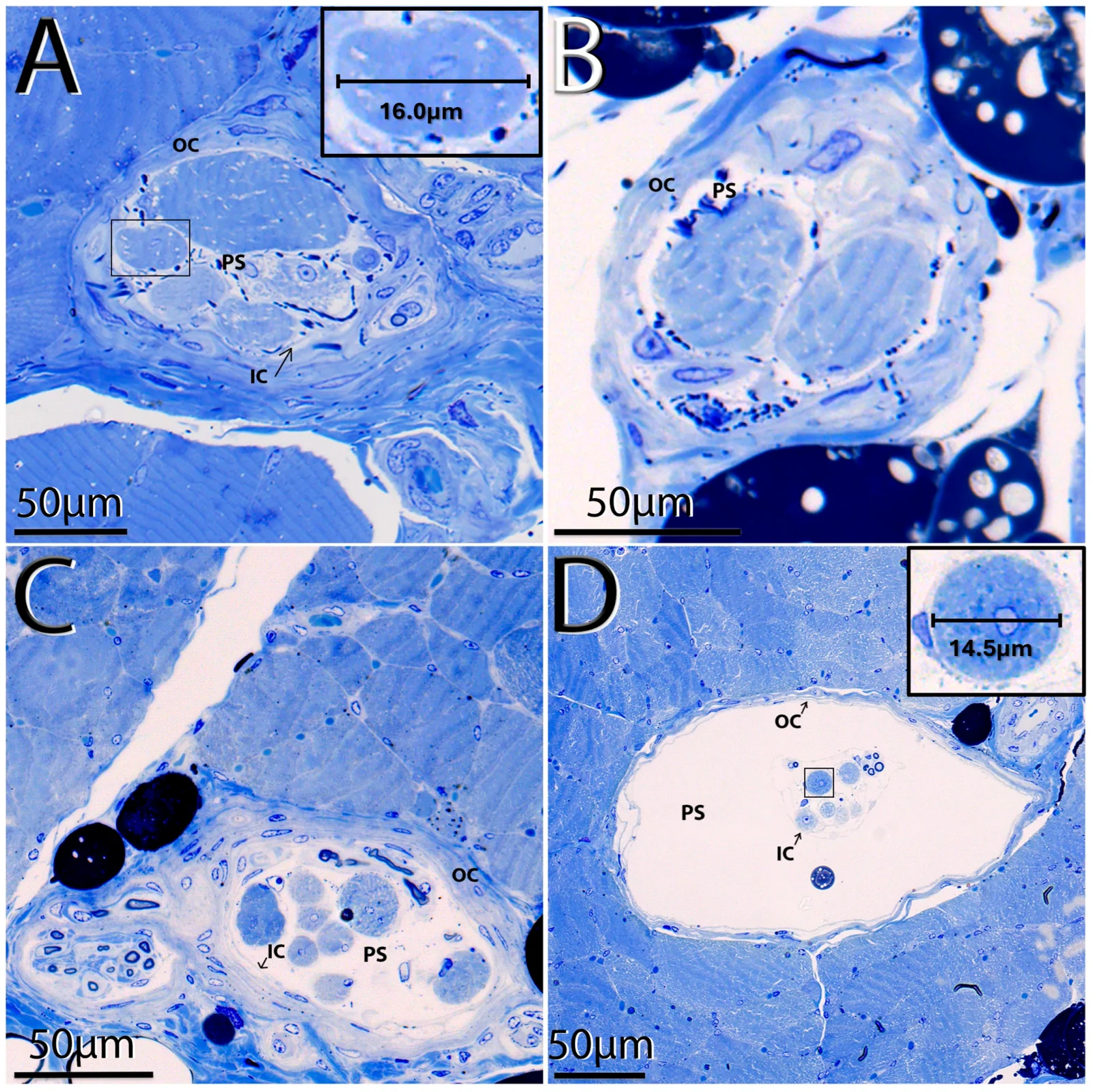
Progressive muscle spindle pathology in DM tibialis cranialis muscle. Toluidine blue-stained cross-sections (allowing for the visualization of myofibers) of muscle spindles from control (**A**), early-stage DM (**B**), and late-stage DM (**C**,**D**). Control and early-stage spindles exhibit preserved intrafusal fiber organization and capsule structure. Late-stage DM spindles demonstrate widening of the periaxial space and pronounced degeneration of the intrafusal muscle fibers. Insets in (**A**,**D**) show diameter of the highlighted myofiber. OC= outer capsule; IC = inner capsule; PS = periaxial space. Micron bars = 50 µm.

Muscle spindle morphology and SOD1 immunoreactivity differed across disease stages (Figure 9 and Figure 10). Per dog, 1–3 spindles were assessed. In control dogs, spindles consisted of tightly packed intrafusal fibers enclosed in the inner capsule, which was in close proximity to the outer capsule layers. Minimal to no SOD1 labeling was noted. Compared to controls, early-stage DM samples appeared to present stronger SOD1 immunoreactivity within intrafusal myofibers and within the spindle capsule. In late-stage samples, more pronounced structural abnormalities were evident, including widening of the space between intrafusal myofibers, a substantially larger periaxial space, and robust SOD1 immunolabeling throughout both the myofibers and the capsule. Particularly marked SOD1 labeling was noted along the internal surface of the outer capsule. Similarly to the heterogeneity observed in extrafusal myofibers, the severity of spindle pathology varied within the same muscle section. In late-stage samples, some spindles remained relatively well preserved, yet still showed higher SOD1 labeling than those from early-stage dogs, whereas other spindles within the same tissue section displayed marked degeneration, with loose intrafusal myofiber packing, pronounced decreases in intrafusal myofiber diameters, and intense SOD1 immunoreactivity (Figure 9 and Figure 10). Masson trichrome staining was used to qualitatively assess collagen distribution within the spindles (Figure 9). In contrast to the progressive collagen deposition observed around extrafusal myofibers, no obvious accumulation of collagen was observed between intrafusal myofibers. Overall, these findings indicate that muscle spindles undergo early degenerative changes characterized by increasing SOD1 immunolabel, disruption of intrafusal fiber organization, intrafusal myofiber atrophy, and progressive compromise of capsule integrity, with the extent of pathology varying across fascicles within a single muscle.

## 4. Discussion

The present study demonstrates that the tibialis cranialis muscle undergoes early and progressive structural alterations in dogs with DM, affecting both extrafusal and intrafusal myofibers. These findings suggest the origin of general proprioceptive ataxia early in the disease progression is likely due in part to primary pelvic limb muscle pathology. The progressive alterations in extrafusal myofiber morphology, reduced packing density, changes in fiber type composition, and increasing collagen deposition, indicating substantial remodeling of the muscle microenvironment as disease progresses, likely contribute to the progressive muscle weakness characteristic of DM. DM-related pathological changes have also been reported in other pelvic limb muscles [14]. The degenerative changes in the muscle spindles also likely contribute to the progressive disease-related general proprioceptive ataxia. In addition to the disease-related changes in the muscle spindles, DM is accompanied by pathology in other parts of the general proprioceptive pathway that collectively contribute to the pelvic limb deficits of affected dogs [11].

Interpretation of myofiber size and fiber type composition in canine skeletal muscle requires consideration of biological variables known to influence muscle phenotype. Fiber type distribution varies among breeds, muscles, and sampling locations [15,16,17,18,19]. Previous studies reported that the tibialis cranialis muscle normally contains approximately 34–36% type I fibers in healthy dogs, consistent with the control dogs in the present study [15,19]. To reduce the variability, all samples were collected from the same anatomical region and exclusively from Pembroke Welch Corgis. Age is another variability to consider, as increased myofiber size variability in older dogs has been reported [16]. No significant age differences were present between groups in this study, suggesting that aging alone does not account for the progressive myofiber variability observed in DM. Sex and reproductive status may also affect skeletal muscle characteristics, as female dogs can exhibit differences in fiber type composition and type II myofiber size following gonadectomy [19,20]. Although some contribution of these factors cannot be excluded, they would not be expected to account for the progressive pathology observed in this study.

The changes observed in extrafusal muscle fiber type composition provide additional insight into disease mechanisms in DM. In contrast to the fiber type grouping typically observed in some forms of ALS after motor neuron degeneration and loss and compensatory reinnervation, DM-affected tibialis cranialis muscles exhibited what appeared to be a progressive reduction in the proportion of type I fibers without clear evidence of fiber type grouping. This pattern is consistent with previous investigations of skeletal muscle pathology in DM, in which fiber type grouping has not been observed in PWCs, yet alterations in fiber type proportions have been reported [4,14,21]. In the extensor carpi radialis muscle, a significant increase in type I to type II fiber ratio was detected in early disease that continued to progress in late stages [21]. Likewise, thoracic intercostal muscles exhibited a marked increase in type I fibers in late-stage disease relative to controls [4]. In contrast, evaluation of the gastrocnemius muscle in PWCs and Boxers reported clear fiber type grouping in Boxers, but not PWCs (details on fiber type proportions were not discussed) [14]. Accumulating evidence from the ALS literature suggests that skeletal muscle exhibits cell-autonomous disease responses, including the ability to undergo metabolic reprogramming in response to altered energy demands [22,23,24,25]. In ALS, this has been demonstrated as a shift in glycolytic (type II) fibers toward increased reliance on fatty acid oxidation [26,27,28], consistent with the increased ratio of type I myofibers seen in intercostal and extensor carpi radialis muscles in DM. In contrast, the preferential reduction in type I fibers observed in the cranial tibialis muscle may reflect a failure of oxidative fibers to compensate under conditions of chronic metabolic and oxidative stress, potentially exacerbated by SOD1-associated toxicity. These findings suggest that fiber type alterations may be influenced by muscle-specific metabolic demands, as well as breed-related differences in disease expression.

The progressive increase in SOD1 immunoreactivity observed in this study further supports the concept that SOD1-associated neurodegenerative disease involves primary pathological processes beyond neurons. In DM-affected dogs, increased SOD1 immunoreactivity was detected not only in the neuronal populations reported in previous studies, but also in multiple muscle-associated cell types, including extrafusal myofibers, intrafusal myofibers, supporting cells, and spindle capsule structures. SOD1 immunoreactivity increased in a stage-dependent manner, transitioning from minimal labeling in control samples to localized labeling in early disease, affecting a subset of fibers, and ultimately to widespread involvement of the majority of myofibers and spindle structures in late-stage DM. This pattern closely parallels previously reported findings in the lumbar dorsal root ganglion (DRG), where early disease is characterized by focal SOD1 immunolabeling within a limited number of neurons, followed by more widespread labeling in advanced stages [11]. The similarities in progression between muscle tissue and DRG sensory neurons suggest that SOD1 pathology may expand within affected tissue over time. Such patterns are consistent with proposed mechanisms of misfolded SOD1 propagation, in which aberrant protein species spread progressively through interconnected cellular environments [29,30,31,32,33]. Overall, these findings reinforce growing evidence that SOD1 accumulation is not restricted to neurons, but involves multiple cell types within affected tissues, highlighting the multiple-system disease of DM. Although SOD1 accumulation is associated with muscle and nerve pathology, the mechanisms underlying this association remain to be determined [34,35].

In addition to myofiber labeling, numerous small cells with strong SOD1 immunoreactivity were seen adjacent to extrafusal myofibers. The identity of these cells was not determined in this study; however, their morphology and distribution are consistent with immune cells that may selectively take up and sequester the variant SOD1. Strong SOD1 labeling was also frequently observed along the outer capsule of the muscle spindles. The capsule is composed of perineural cells, specialized flattened epithelioid myofibroblasts with high metabolic activity that form a selective blood-spindle barrier [36], raising the possibility that these structures may be particularly susceptible to SOD1-associated pathology.

Although general proprioceptive ataxia is known to contribute to the earliest clinical signs of DM, muscle spindles, the receptor structures for proprioception, have not been previously evaluated for disease-related pathology. The absence of overt structural degeneration in early-stage spindles suggests that functional alterations may precede detectable morphological changes. Alternatively, these findings raise the possibility that muscle spindle pathology may not be a primary driver of the early proprioceptive deficits observed in DM, but rather reflects later-stage involvement of peripheral sensory structures as disease progresses. Pathology in other components of the general proprioceptive pathways almost certainly contributes to early functional deficits [11]. In control dogs, spindles display tightly packed intrafusal fibers enclosed within a well-defined capsule. In early-stage DM, overall spindle architecture remained largely preserved, whereas in late-stage disease, more pronounced structural abnormalities were evident, including widening of the space between and pronounced decreases in diameters of intrafusal fibers, as well as swelling of the spindle capsule. It is unclear whether the increased periaxial space represents true disease-related alterations or reflects variation in the anatomical level at which the spindle was sectioned within its three-dimensional structure. Further studies involving serial sectioning through entire spindles would be necessary to determine the extent to which these observations represent consistent pathological features. Collectively, these observations highlight the need for deeper investigation into muscle spindle architecture in DM to clarify the nature and functional implications of histopathological changes.

These observations can be considered in the context of previously reported degeneration within central and peripheral proprioceptive pathways. Although DM does not appear to entail overt axonal loss from the lumbar sensory roots, substantial pathology has been documented within the dorsal and ventral spinocerebellar tracts and the fasciculus gracilis [11,13]. Early-stage disease is associated with decreased axon density and myelin abnormalities within these spinal sensory pathways, while later stages show further alterations in axon diameter and myelination. These findings indicate that proprioceptive circuits are affected early in DM. The spindle alterations observed in the present study, therefore, extend prior observations of sensory pathway degeneration at the level of the peripheral receptor, suggesting that multiple components of the proprioceptive system become involved as the disease advances.

The observations reported in this study share several important parallels with the skeletal muscle pathology described in ALS. Muscle biopsies from ALS patients frequently demonstrate features of chronic neurogenic pathology, including generalized atrophy and atrophic fibers [26,27]. In addition to these apparent neurogenic changes, several studies have reported myopathic features, such as increased fiber size variability, internalized nuclei, and degenerative changes within muscle fibers, suggesting that direct skeletal muscle pathology may play an active role in ALS [26,28,37]. Progressive remodeling of the muscle microenvironment, including extracellular matrix deposition and fibrosis, is also recognized as a contributor to functional decline in this disease [38].

Similar patterns were observed in the present study in dogs affected by DM. In addition to changes consistent with denervation, some late-stage DM muscles exhibited more generalized myofiber atrophy that resembled age-related sarcopenia. Comparable observations were previously reported in the gastrocnemius muscle of late-stage DM-affected PWCs, where widespread myofiber atrophy occurred in the absence of fiber type grouping, leading the authors to suggest a contribution of accelerated sarcopenia in addition to denervation-related pathology [14]. Age-related skeletal muscle changes in dogs are characterized by increased variability in fiber size, selective myofiber atrophy, and progressive loss of lean muscle mass, even in the absence of primary neuromuscular disease [16,39,40,41]. Notably, the ages of the DM-affected and control dogs in the present study were not significantly different, suggesting that the generalized atrophic changes observed in the affected animals are unlikely to reflect normal aging alone. Instead, DM may accelerate or exacerbate age-associated muscle remodeling, resulting in a mixed pattern of neurogenic and sarcopenia-like changes. Although additional histochemical and molecular analysis would be required to definitively distinguish these processes, the findings further support the concept that skeletal muscle pathology in DM is more than a consequence of motor neuron pathology. Because nerve and muscle pathology occur concurrently in DM and *SOD1*-associated ALS, it is not possible to determine definitively whether muscle pathology is secondary to neural degeneration or vice versa in vivo. However, cell culture experiments demonstrated that myotubes derived from ALS patients release extracellular vesicles, the contents of which are toxic to motor neurons, suggesting that primary changes in muscle cells can lead to secondary neuropathology in *SOD1*-associated ALS and DM [42]. In addition, it was found that, in a mouse model, selective expression of mutant SOD1 in skeletal muscle resulted in progressive muscle atrophy and marked neuromuscular junction abnormalities, along with formation of ubiquitinated inclusions in spinal cord motor neurons and degeneration of these neurons [25]. This indicates that skeletal muscle pathology can occur as a direct result of endogenous expression of mutant SOD1 and that affected muscle can be a source of SOD1 toxicity to innervating neurons. The potential that muscle pathology in DM and some forms ALS is a direct result of mutant SOD1 expression in muscle needs to be considered in developing approaches to therapeutic interventions.

While skeletal muscle (extrafusal myofiber) pathology has been relatively well described in ALS, comparatively little attention has been given to the structural integrity of muscle spindles, especially in cases with clinical sensory deficits. Nevertheless, emerging evidence suggests that proprioceptive pathways associated with muscle spindles may be affected in some forms of ALS. In a mouse model carrying a mutation in *SOD1*, pathological changes in Ia/II proprioceptive nerve endings at muscle spindles occurred early and may precede overt motor neuron degeneration [43]. Similarly, misfolded SOD1 has also been detected in large proprioceptive neurons of the dorsal root ganglia (DRG), where it was associated with neuronal degeneration and inflammatory responses [44]. Histopathological investigations in human ALS patient tissue described abnormalities in intramuscular nerve fibers associated with muscle spindles, including alterations in gamma axons and other intrafusal nerve structures [45]. While rodent models do not fully recapitulate the complexity and clinical heterogeneity of human ALS, these studies collectively highlight a significant gap in our understanding of proprioceptive involvement in ALS disease pathogenesis and underscore the need for investigation in complementary translational models. The naturally occurring DM dog model provides a valuable opportunity to evaluate muscle spindle pathology in a large-animal *SOD1*-associated disease model and may help clarify the contribution of proprioceptive structures to disease progression.

## 5. Conclusions

Overall, the findings of this study expand current knowledge of skeletal muscle pathology in DM by demonstrating that disease-related changes involve both extrafusal and intrafusal fibers and muscle spindles. The presence of early and progressive spindle pathology is particularly notable, as these structures represent a critical peripheral interface linking skeletal muscle to central proprioceptive pathways. By documenting pathological changes across multiple muscle components and disease stages, this study further supports the concept that DM represents a multiple-system disorder. Importantly, the similarities observed between DM and ALS further strengthen the value of DM as a naturally occurring translational model for some forms of *SOD1*-associated ALS. Continued investigation of both motor and sensory elements of the neuromuscular system in DM may therefore provide important insights into disease mechanisms and potential therapeutic targets relevant to human ALS. The muscle pathology characterized in this study suggests that assessment of potential therapeutic interventions for *SOD1*-associated ALS will need to include consideration of directly targeting muscle in addition to sensory and motor neurons. A limitation of this study is the relatively small sample sizes, due to challenges in recruiting samples from privately owned dogs.

## Figures and Tables

**Table 2:** Signalment summary of dogs included in this study.

Identification #	DM Disease Stage	*SOD1* Genotype	Age (yr)	Sex	Neuter Status
403	0	A/A	10.8	F	S
438	0	A/A	14.0	F	S
443	0	A/A	13.5	F	S
433	0	A/G	15.8	F	---
423	0	A/A	15.1	F	S
407	1	A/A	13.0	F	S
453	2	A/A	Unknown	M	C
452	2	A/A	13.3	M	C
463	2	A/A	12.1	M	C
454	2	A/A	12.6	F	S
458	2	A/A	12.3	F	S
428	3	A/A	11.8	M	C
457	3	A/A	13.9	F	S
439	3.5	A/A	13.5	M	C
427	4	A/A	13.8	F	---
445	4	A/A	11.0	F	---
446	4	A/A	12.8	M	C
430	4	A/A	11.5	M	C
426	4	A/A	15.6	M	C

F = Female. M = Male. S = Spayed. C = Castrated. --- = Intact.

## Data Availability

The original contributions presented in this study are included in the article. Further inquiries can be directed to the corresponding author.

## Supplementary Materials

The following supporting information can be downloaded at: https://www.mdpi.com/article/10.3390/ani16182817/s1, Supplemental File S1: Tissues Collected from Dogs Utilized in the Study; Supplemental File S2: Masson Trichrome Staining Protocol.

## Abbreviations

The following abbreviations are used in this manuscript:

DM	Degenerative myelopathy
ALS	Amyotrophic lateral sclerosis
SOD1	Superoxide dismutase 1
PWC	Pembroke Welch Corgi
MHC-1	Myosin heavy chain-1
OC	Outer capsule
IC	Inner capsule
PS	Periaxial space
DRG	Dorsal root ganglion
