# Supplementary material for: Tibialis Cranialis Extrafusal and Intrafusal Myofibers and Muscle Spindle Pathology in Canine Degenerative Myelopathy
Source: Animals (Basel). 2026 Sep 8;16(18):2817. doi: 10.3390/ani16182817 (PMC13603699; doi:10.3390/ani16182817)
Supplement: Supplementary file 1 [file animals-16-02817-s001.zip › Supplemental File S1.pdf]

Supplemental File 1

Tissues Collected from Dogs Utilized in the Study

Tissues used in these studies were collected from privately owned dogs at the time of euthanasia with the informed consent of the owners. Tissue collections were performed at the University of Missouri for dogs that were euthanized locally and by veterinarians at other locations where the dogs were euthanized. Tibialis cranialis, thoracic spinal cord, and blood samples were obtained from all dogs evaluated for this study. Additional tissue samples were obtained from some of the dogs for studies on other aspects of the disorder. For all of the dogs euthanized at the University of Missouri, all of the samples listed below were obtained. Due to logistical limitations, some of the tissues not required for this study were not obtained from some of the dogs euthanized elsewhere.

<u>Complete List of Tissues Collected Locally</u>

- Blood sample in purple-top tube for SOD1 genotyping.
- Fixed thoracic spinal cord for histopathological confirmation of disease status.
- Tibialis cranialis muscles samples
  - Fixed in immunology fixative
  - Fixed in glutaraldehyde fixative
  - Preserved in cacodylate buffer

<u>Additional tissues collected from locally euthanized dogs and some dogs euthanized elsewhere for other related studies</u>

- C3 Dorsal Root Ganglion (DRG) (Formalin)
- C8 Cord with DRG (Formalin)
- T4 Cord with DRG (Formalin)
- T7 Cord with DRG (Formalin)
- T9 Cord with DRG (Formalin)
- L2 Cord with DRG (Formalin)
- L3 DRG (Formalin)
- Skin biopsy (caudal medial thigh) (Formalin)
- Sural Nerve (Formalin)
- Sciatic Nerve (Formalin)
- Fibular Nerve (Formalin)
- Ulnar Nerve (Formalin)

- Femoral Nerve (Formalin)
- Biceps femoris (Formalin)
- Ext Carpi Radialis (Formalin)
- Ext Digit Brevis m (Distal) (Formalin)
- Half brain (Formalin)
- Tongue (Formalin)
- C4 Cord with 1 DRG (Immuno Fix)
- C4 DRG (Immuno Fix)
- DMND-509_C5 DRG (Immuno Fix)
- T1 Cord with DRG (Immuno Fix)
- T5 Cord with DRG (Immuno Fix)
- T10 Cord with DRG (Immuno Fix)
- L3 Cord with 1 DRG (Immuno Fix)
- Biceps femoris (Immuno Fix)
- R. Ext Digit Brevis m. (Distal) (Immuno Fix)
- Ext Carpi Radialis (Immuno Fix)
- Left eye cup with optic nerve (Immuno Fix)
- Right eye cup with optic nerve (Immuno Fix)
- C3 Cord with 1 DRG (EM Fix)
- C7 Cord with DRG (EM Fix)
- T8 Cord with DRG (EM Fix)
- Cord with DRG (EM Fix)
- Sural Nerve (EM Fix)
- Sciatic Nerve (EM Fix)
- Fibular Nerve (EM Fix)
- Ulnar Nerve (EM Fix)
- Femoral Nerve (EM Fix)
- Biceps Femoris (EM Fix)
- L. Ext Digit Brevis m (Distal) (EM Fix)
- Ext Carpi Radialis (EM Fix)
