# Supplementary material for: Tibialis Cranialis Extrafusal and Intrafusal Myofibers and Muscle Spindle Pathology in Canine Degenerative Myelopathy
Source: Animals (Basel). 2026 Sep 8;16(18):2817. doi: 10.3390/ani16182817 (PMC13603699; doi:10.3390/ani16182817)
Supplement: Supplementary file 1 [file animals-16-02817-s001.zip › Supplemental File S2.pdf]

Supplemental File 2

Masson Trichrome Staining Protocol

(Adapted from Sigma-Aldrich Procedure No. HT15)

1. Embed fixed tissue sample in paraffin.
2. Cute sections at 0.4 um and mount on Fiser Superfrost slides.
3. Deparaffinize slides and hydrate to deionized water.
4. Incubate in Bouin's Solution at 56°C for 15 minutes.
5. Wash slides in running water at room temperature to remove yellow color from sections.
6. Incubate in Weigert's Iron Hematoxylin Solution for 5 minutes.
7. Rinse in deionized water.
8. Incubate in Biebrich Scarlet-Acid Fucshin for 5 minutes.
9. Rinse in deionized water.
10. Incubate in Phosphotungstic/Phosphomolybdic Acid Solution for 5 minutes.
11. Incubate in in Aniline Blue Solution for 5 minutes.
12. Incubate in 1% acetic acid for 2 minutes.
13. Rinse slides in deionized water, dehydrate through alcohol, clear in xylene and mount coverslip.
